# Clear Cell Variant of Oral Squamous Cell Carcinoma: A Rare Case

**DOI:** 10.7759/cureus.77450

**Published:** 2025-01-14

**Authors:** Ananjan Chatterjee, Swapan K Purkait, Hiralal Ash, Ishita Banerjee, Abhishek Banerjee, Karthikeyan Ramalingam

**Affiliations:** 1 Oral and Maxillofacial Pathology, Buddha Institute of Dental Sciences and Hospital, Patna, IND; 2 Oral and Maxillofacial Surgery, Buddha Institute of Dental Sciences and Hospital, Patna, IND; 3 Pediatric and Preventive Dentistry, Guru Nanak Institute of Dental Sciences and Research, Kolkata, IND; 4 Oral and Maxillofacial Pathology, Awadh Dental College and Hospital, Jamshedpur, IND; 5 Oral Pathology and Microbiology, Malla Reddy Institute of Dental Sciences, Malla Reddy Vishwavidyapeeth, Hyderabad, IND

**Keywords:** clear cell, cytokeratin 8/18, cytokeratin ae1/ae3, immunohistochemistry, malignancy, mandible, neoplasm, oral squamous cell carcinoma, retromolar region, right side

## Abstract

The clear cell variant of oral squamous cell carcinoma (CCSCC) is an uncommon aggressive lesion found in the oral cavity. Limited cases of CCSCC have been documented in the literature. This report discusses a 38-year-old female presenting with a non-healing ulcer in the right retromolar region, accompanied by pain and discomfort. Histopathological analysis revealed sheets and islands of severely dysplastic epithelial cells with clear cytoplasm and keratin pearls within the connective tissue, indicating a rare variant of oral squamous cell carcinoma. Various differential diagnoses were kept in mind, and a series of histopathological, histochemical, and immunohistochemical markers were carried out to confirm the origin of these clear cells. It was positive for cytokeratin 8, cytokeratin 18, and cytokeratin cocktail (AE1/AE3). It was negative for vimentin, S100, and HMB45. Thus, our final diagnosis was a CCSCC. This variant is seldom encountered. It often presents diagnostic challenges and is also an enigma to understanding its biological behavior.

## Introduction

Only 3% of all malignancies are head and neck, and more than 90% are oral squamous cell carcinomas [1,2]. Most commonly, they are histopathologically graded as well-differentiated, moderately differentiated, and poorly differentiated lesions based on their keratinization and dysplasia. Other rare histopathological variants of oral squamous cell carcinoma include verrucous, spindle cell, adenosquamous, basaloid, and clear cell variants [2].

Clear cell squamous cell carcinoma, a rare variant of squamous cell carcinoma of the skin, was first identified by Kuo in 1980; squamous cell carcinoma involving the oral cavity with clear cell changes is even rarer. Only a few cases have been reported [1-3]. Researchers propose that clear cell transformation may occur due to hydropic degeneration of neoplastic cells and could be due to the accumulation of mucin, glycogen, intracellular fluids, or lipids [1,3].

Here, we report a rare case of the clear cell variant of oral squamous cell carcinoma (CCSCC) involving the right retromolar trigone in a middle-aged female.

## Case presentation

A 38-year-old female patient was referred with complaints of severe pain in the posterior lower jaw and chewing difficulty for the past 10 days. She reported a history of chewing non-smoking tobacco for the past 20 years. Her past medical history, surgical history, and dental history were noncontributory.

**Figure 1:**
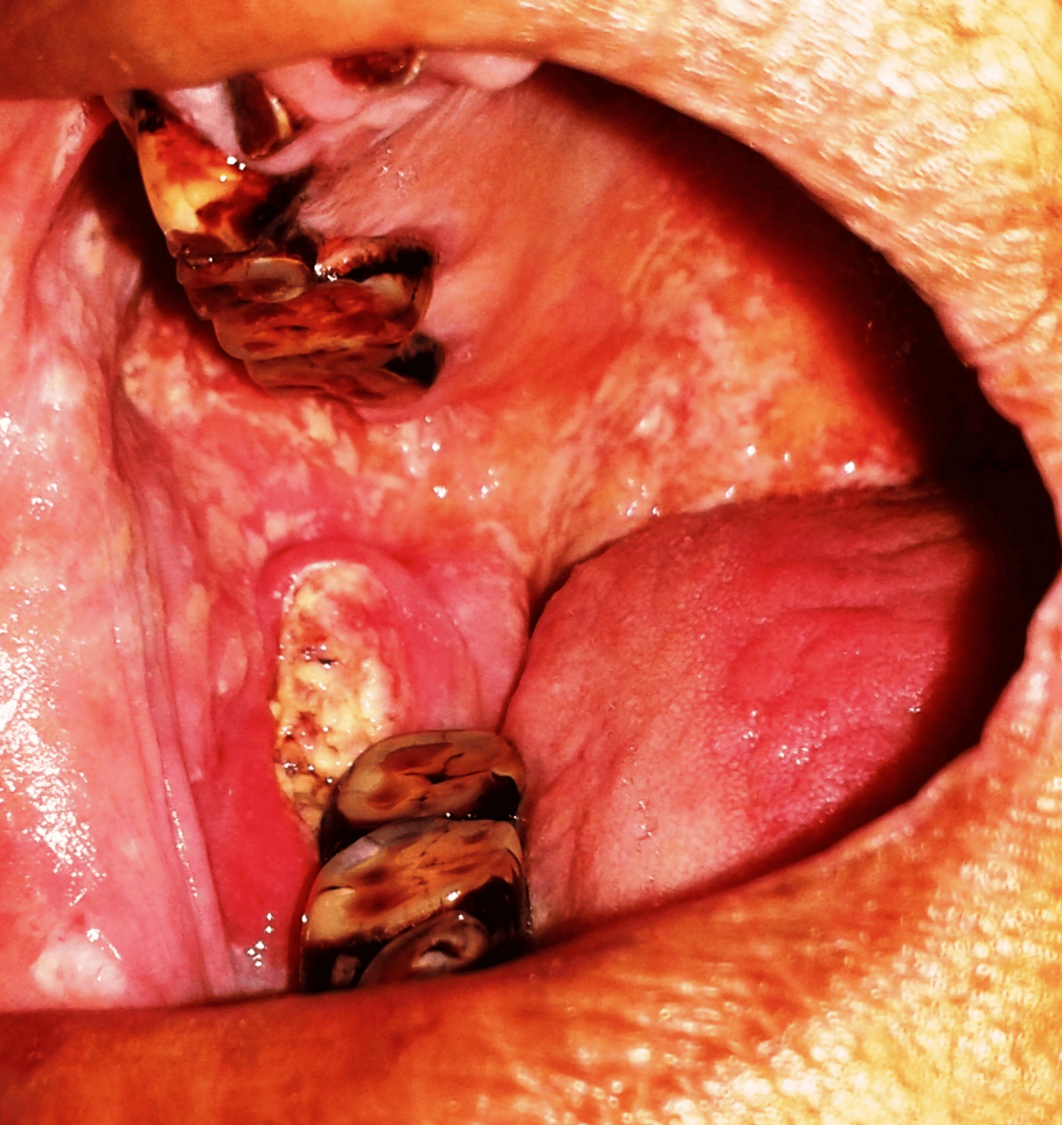
Clinical picture showing the ulceroproliferative lesion on the right retromolar trigone

The extraoral examination was normal. Intraoral examination revealed an ulceroproliferative growth with non-scrapable peripheral mixed white and erythematous areas involving the right retromolar trigone region. It was approximately 2.5 cm in the anteroposterior dimension and 3 cm in the superoinferior dimension. It was well-defined on the posterior margin but ill-defined in the antero-inferior region (Figure 1).

The orthopantomogram did not show any significant changes. An incisional biopsy was performed, and the tissue specimen was submitted for histopathological examination.

**Figure 2:**
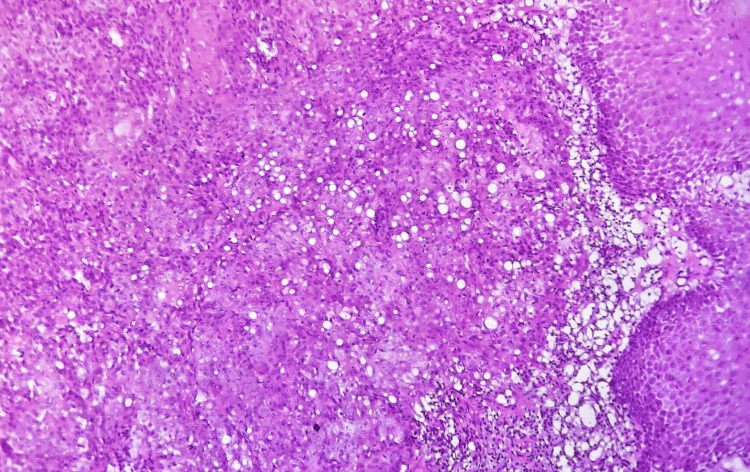
Photomicrograph showing malignant epithelial islands in the stroma along with numerous clear cells (H&E, 10x) H&E: hematoxylin and eosin

Histological sections revealed dysplastic epithelium with focal areas of ulceration and breach in the continuity of the basement membrane. Malignant epithelial cells invaded the superficial stroma in the form of multiple islands. Clear cells, which were round to ovoid in shape with clear cytoplasm and varying degrees of nuclear pleomorphism, were noted amid the malignant epithelial cells. Focal areas of necrosis and hemorrhage were also evident (Figure 2).

**Figure 3:**
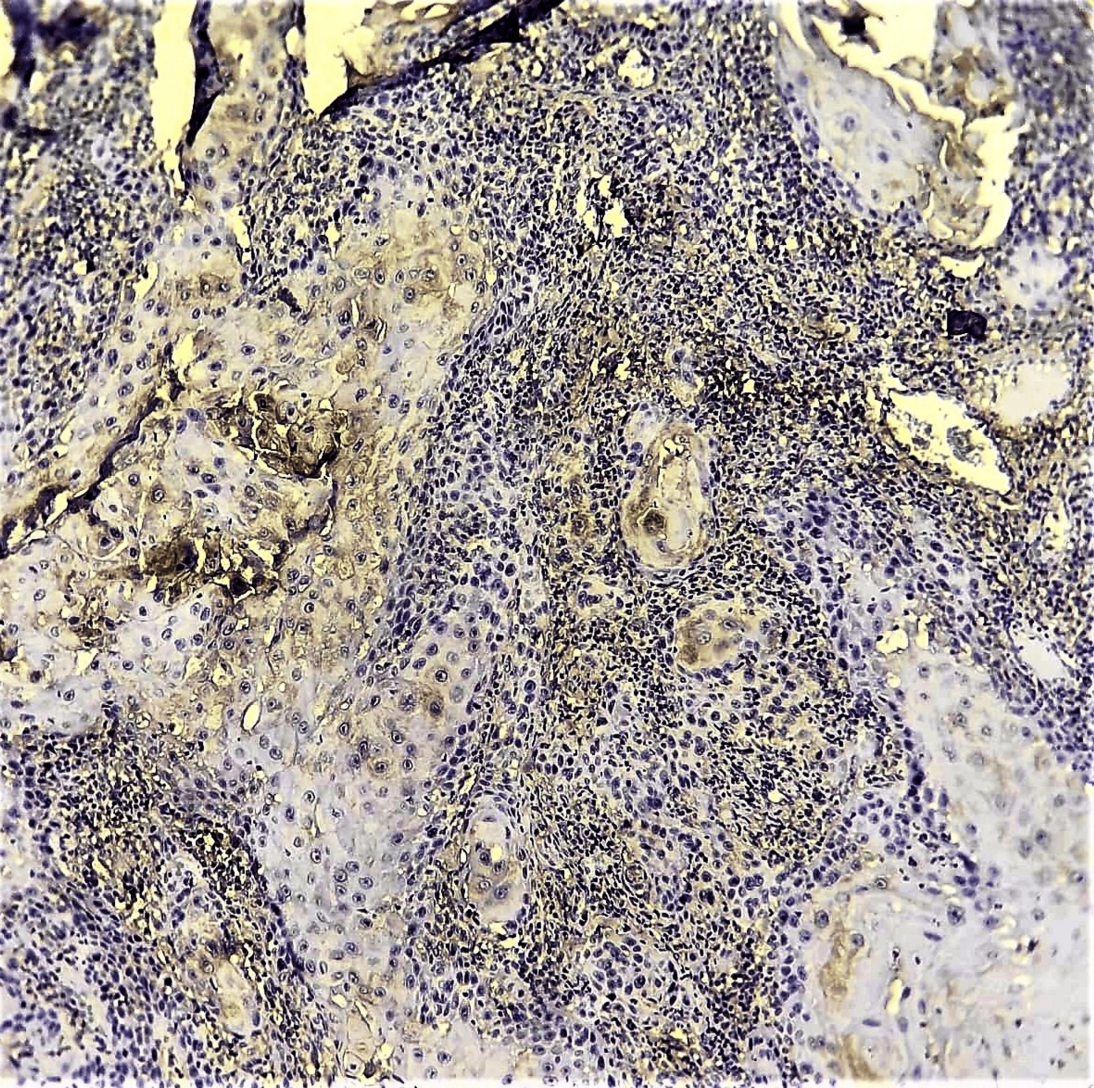
Photomicrograph showing focal positivity to CK8/CK18 (IHC, 10x) IHC: immunohistochemistry

Special stains for mucin and immunohistochemical (IHC) investigations were carried out to rule out salivary gland malignancies or odontogenic epithelial origin. Periodic acid-Schiff (PAS) and mucicarmine staining yielded negative results. IHC showed strong immunopositivity for CK8/CK18 (Figure 3) and AE1/AE3 while negative for vimentin, HMB45, and S100.

A final diagnosis of the CCSCC was established based on histopathological and IHC observations. The patient was referred to the regional cancer institute for further management, but follow-up after a few months revealed that the patient had not initiated the recommended therapy.

## Discussion

The CCSCC is an extremely rare form of oral squamous cell carcinoma. Clear cell alterations are attributed to hydropic degeneration of neoplastic cells, leading to intracellular fluid accumulation, and could be associated with glycogen, lipid, intracellular fluid, or mucin accumulation. Therefore, researchers also termed this variant hydropic squamous cell carcinoma [2-4].

Clear cells are frequently observed in advanced squamous cell carcinoma cases, representing either a secondary phenomenon or clonal development. While the WHO has classified CCSCC as a distinct entity in areas such as the skin and penis, it has not yet been recognized as such in the head and neck region. These tumors are typically aggressive [4]. According to the literature, CCSCC is mostly observed in cutaneous areas. However, cases have been reported in the alveolar ridge, buccal mucosa, and tongue of the oral cavity. Clinically, it commonly presents as a nodule or mass that may occasionally ulcerate [2]. Possible etiological factors include immunosuppression, arsenic exposure, radiation, and chronic ulceration [5]. Hirose et al. [6] directly linked the CCSCC to genetic mutations in PIK3CA p.E542K (c.1624G>A) and HRAS p.G12A (c.35G>C), highlighting the significance of these oncogenes as potential therapeutic targets [6,7].

The literature classifies CCSCC into three major types: keratinizing, non-keratinizing, and pleomorphic types 1, 2, and 3, respectively. Type I tumors are composed of neoplastic cells arranged in sheets or islands, characterized by clear, "bubbled" cytoplasm, areas of keratinization, and the formation of keratin pearls. Type II tumors are primarily skin neoplasms, and they display anastomosing cords of clear cells with centrally located nuclei that lack keratinization or glandular differentiation. Type III tumors demonstrate marked pleomorphism with extensive vascular and perineural invasion [3,8].

In our case, the patient had a history of chewing non-smoking forms of tobacco, and it is presumed that the lesion evolved from a pre-malignant lesion in the retromolar region. Clear-cell tumors are mostly odontogenic or salivary gland or metastatic in origin. A thorough IHC panel could be performed to exclude these possibilities [4]. Differential diagnoses included clear-cell odontogenic carcinoma, which was ruled out as PAS staining was negative, indicating the absence of glycogen. Additionally, the presence of severe malignant characteristics and the absence of stromal calcifications excluded the possibility of calcifying epithelial odontogenic tumor clear cell variant. Salivary gland neoplasms, such as epithelial-myoepithelial carcinoma and clear-cell acinic cell carcinoma or mucoepidermoid carcinoma, could be ruled out due to negative PAS and mucicarmine staining. Negative results for S100 can exclude neural-origin tumors. Amelanotic melanoma accounts for 1% of oral melanomas, and IHC and full-body examination could exclude metastatic renal cell carcinoma [2,3,8,9].

Oral squamous cell carcinoma greatly impacts quality of life [10], and most patients reported greater difficulties with oral functions [11]. Less than 10 cases of CCSCC are reported in English literature [12], and their clinical impact or prognosis is unclear. Localized advanced oral cancer could be managed with a combination of surgery, chemotherapy, and radiotherapy. Metronomic chemotherapy could also be employed for better patient tolerance [13]. Controversy still exists on the pathogenesis of these clear cell variants. The suggestion is that clear cell change could be a part of the acantholytic process in squamous cell carcinoma. Most reported literature suggests a poor prognosis [14], and further clinicopathological information is needed to decipher the course of this rare variant of a commonly reported oral malignancy.

## Conclusions

CCSCC is an exceedingly rare entity posing diagnostic challenges. Comprehensive knowledge of its occurrence, clinical features, and immunoreactivity is essential for accurate diagnosis. The prognosis of CCSCC remains unclear due to the limited cases. Further research will help clinicians with their prognostic outcomes.
